# Assessment of intraductal carcinoma in situ (DCIS) using grating-based X-ray phase-contrast CT at conventional X-ray sources: An experimental ex-vivo study

**DOI:** 10.1371/journal.pone.0210291

**Published:** 2019-01-09

**Authors:** Karin Hellerhoff, Lorenz Birnbacher, Anikó Sztrókay-Gaul, Susanne Grandl, Sigrid Auweter, Marian Willner, Mathias Marschner, Doris Mayr, Maximilian F. Reiser, Franz Pfeiffer, Julia Herzen

**Affiliations:** 1 Institute for Clinical Radiology, Ludwig-Maximilians-University Hospital Munich, Munich, Germany; 2 Abteilung für Diagnostische Radiologie, Rotkreuzklinikum München, Munich, Germany; 3 Chair of Biomedical Physics, Department of Physics & Munich School of BioEngineering, Technical University of Munich, Garching, Germany; 4 Institute of Pathology, Ludwig-Maximilians-University Hospital Munich, Munich, Germany; 5 Institute of Diagnostic and Interventional Radiology, Klinikum rechts der Isar, Technical University of Munich, Munich, Germany; A.C. Camargo Cancer Center, BRAZIL

## Abstract

**Background:**

The extent of intraductal carcinoma in situ (DCIS) is commonly underestimated due to the discontinuous growth and lack of microcalcifications. Specimen radiography has been established to reduce the rate of re-excision. However, the predictive value for margin assessment with conventional specimen radiography for DCIS is low. In this study we assessed the potential of grating-based phase-contrast computed tomography (GBPC-CT) at conventional X-ray sources for specimen tomography of DCIS containing samples.

**Materials and methods:**

GBPC-CT was performed on four ex-vivo breast specimens containing DCIS and invasive carcinoma of non-specific type. Phase-contrast and absorption-based datasets were manually matched with corresponding histological slices as the standard of reference.

**Results:**

Matching of CT images and histology was successful. GBPC-CT showed an improved soft tissue contrast compared to absorption-based images revealing more histological details in the same sections. Non-calcifying DCIS exceeding the invasive tumor could be correlated to areas of dilated bright ducts around the tumor.

**Conclusions:**

GBPC-CT imaging at conventional X-ray sources offers improved depiction quality for the imaging of breast tissue samples compared to absorption-based imaging, allows the identification of diagnostically relevant tissue details, and provides full three-dimensional assessment of sample margins.

## Introduction

In population based mammography screening, intraductal carcinoma in situ (DCIS) represents approximately 20% of detected breast cancers. The extent of DCIS is commonly underestimated due to the discontinuous growth and lack of microcalcifications. As additionally systemic therapy is not recommended in the majority of cases, a DCIS focused management of margins is necessary [1]. Recent studies underline that complete resection in breast conservation during primary surgery for DCIS is mandatory to avoid subsequent surgery and to reduce the risk of recurrent disease [2–5]. Wider resection margins and intraoperative specimen radiography have been established for intraoperative margin assessment to keep the rate of re-excision low. However, the sensitivity and negative predictive value of two-view specimen digital radiography for DCIS is currently low [6]. Pathological methods like intraoperative frozen sections and imprint cytology perform well, but are time consuming and restricted to individual sections of the excised sample [7]. Because of these challenges several X-ray attenuation-based imaging technologies including specimen tomosynthesis specialized for the intraoperative margin assessment are currently under evaluation [8, 9].

Over the last decade, continuous advances in X-ray phase-contrast imaging have rendered the method promising as it provides high soft tissue contrast that substantially exceeds absorption contrast [10]. Initial feasibility trials introduced breast phase-contrast imaging for mammography and CT imaging setups and have been performed with highly brilliant synchrotron sources [11–15].

Compared to phase–contrast mammography, there is little experience with clinical phase-contrast CT applications for breast imaging [16–24]. Recent trials on ex-vivo PC-CT imaging of breast samples using synchrotron radiation showed greatly improved soft tissue contrast and differentiability of fine structures compared to absorption-based imaging [25–30]. The cornerstone for future clinical applications has been advancing grating-based phase-contrast imaging to conventional laboratory X-ray sources [31, 32]. Moreover, grating-based phase-contrast computed tomography (GBPC-CT) provides quantitative tissue-specific values comparable to the Hounsfield units established for conventional CT [33–35].

First ex-vivo breast sample studies using conventional X-ray sources showed improved spatial resolution for the characterization of different breast lesion types, and a full three-dimensional view of a tumor permitting the identification of diagnostically relevant tissue sections within large tumors [36, 37]. A study using synchrotron radiation showed that corresponding to histopathological sections, specific microscopic structures of DCIS can be visualized in PC-CT but not in absorption CT [38].

In this first proof-of-concept study, we perform for the first time ex-vivo grating-based phase-contrast computed tomography (GBPC-CT) using a conventional X-ray source to assess specific imaging features of DCIS compared to histopathology. In detail, we investigate if the specific value of enhanced contrast in grating-based high resolution PC-CT can be compared to absorption-based CT by means of four ex-vivo samples containing NST and DCIS.

## Materials and methods

### Study design

This prospective ex-vivo study was conducted in accordance with the Declaration of Helsinki and was approved by the institutional review board. Two participants were included, giving written informed consent after adequate explanation of the study protocol. Indication to breast surgery followed the recommendation of the interdisciplinary tumor conference of the local certified breast center. Inclusion criteria were a histologically proven invasive or intraductal carcinoma in situ (DCIS) in preoperative biopsy and completed preoperative conventional breast diagnostics (clinical breast examination, high-frequency ultrasound, and digital two-view mammography).

Patient 1 presented with a palpable mass in the upper outer quadrant of the right breast. Mammography revealed multicentric disease with two suspect lesions in the upper outer quadrant and an area of suspect, pleomorphic microcalcifications with segmental distribution in the center of the right breast. Patient 2 presented with inflammatory signs and an induration of the entire left breast. Mammography revealed an edema of the cutis and extensive microcalcifications of both upper quadrants extending into the lower quadrants. Both patients were treated by modified radical mastectomy carried out by the local department of gynecology and obstetrics.

### Sample acquisition and preparation

Within one hour after ablation, the breast abladates were fixated in a 4% formaldehyde solution. Clinical standard histopathological workup was completed before acquisition of the GBPC-CT images. After cutting the formaldehyde-fixated abladates into 5 mm thick slices, macroscopically suspicious and representative tissue sections (max. 3.0 × 2.0 × 0.5 cm^3^) were resected for standard paraffin embedding and automatic staining. For imaging purposes, representative and orientable tissue sections of 3 cm maximum diameter were resected from the tumor-bearing area and put into a 50 ml plastic container containing a 4% neutral-buffered formaldehyde solution. Standard histopathological workup of these samples was performed after PC-CT data acquisition. The slices were dehydrated in an ascending alcohol series before embedding in hot paraffin wax. After solidification, the paraffin blocks were cut into 5 μm sections using a standard microtome and sections were stained with haematoxylin and eosin using standard protocols. Four breast samples were analyzed: three samples from patient 1 and one sample from patient 2.

### Grating-based PC-CT

Grating-based phase-contrast computed tomography (GBPC-CT) uses a conventional X-ray source in combination with a Talbot-Lau interferometer and an X-ray detector. GBPC-CT provides three complementary signals: the conventional attenuation image, the differential phase-contrast signal, and the dark-field image. The basic principles of grating-based phase-contrast imaging can be found in Pfeiffer et al. [31, 32].

The experimental setup consisted of a rotating anode with a molybdenum target (Enraf Nonius FR 591) operating at 40 kVp and 70 mA and a Pilatus II 100k (DECTRIS, Baden, Switzerland) single-photon counting detector (1 mm silicon sensor, 487 × 195 pixels, 172 × 172 μm^2^ pixel size). Due to the sample magnification, the effective pixel size was 100 × 100 μm^2^ [39].

The Talbot-Lau interferometer consists of three gratings, each with a grating period of 5.4 μm (Institut für Mikrostrukturtechnik, Karlsruhe Institute of Technology, Germany) The gratings are placed in equal distances of 85.7 cm. The source grating produces an array of partially coherent X-ray sources and allows for the use of a conventional X-ray tube. The phase grating induces an interference pattern in certain distances exploiting the Talbot effect. The design energy for the phase-grating is 27 keV and the phase-shift is *π* for this energy. The analyzer grating is needed in order to resolve the interference pattern as the detector pixel size is larger than the grating period. A discrete, lateral shift of the analyzer grating over several (phase-) steps called phase-stepping enables the retrieval of the attenuation and differential phase-shift signal of the examined object in the X-ray beam [40]. The number of phase-steps in this study was 11 and the exposure time was 3 seconds per step. The visibility, which describes the quality of the interferometer, was approximately 24%. The reconstruction of the 800 projections for each image signal over 360 degrees was performed via filtered-backprojection using a Ram-Lak filter kernel for the attenuation projections and a Hilbert filter for the differential phase-contrast projections [32]. The sample was positioned directly in front of the phase-grating and surrounded by a water bath to avoid phase-wrapping and to reduce the effect of beam hardening, which facilitates quantitative imaging [35]. The phase-contrast and attenuation data was converted to attenuation Hounsfield units (HU) or phase-contrast Hounsfield units (HUp), respectively [35]. Additionally, the raw detector data were deconvolved using a Richardson-Lucy algorithm with an experimentally determined point-spread function with 10 iterations, in order to correct the image blurring by a large source size [41, 42]. Absorption contrast and PC-CT data were automatically co-registered and manually matched with corresponding histological slices. Preliminary three-dimensional reconstructions of the CT data sets were then matched during a consensus meeting of the reconstructing breast radiologist and the pathologist in a lesion by lesion manner. Although simultaneously acquired in GBPC-CT, the three-dimensional reconstruction of the dark-field signal has not been evaluated with respect to breast imaging in this study.

## Results and discussion

The GBPC-CT data was successfully matched with corresponding histological sections based on characteristic macroscopic features and distribution of adipose tissue.

### Tumor 1

**Fig 1:**
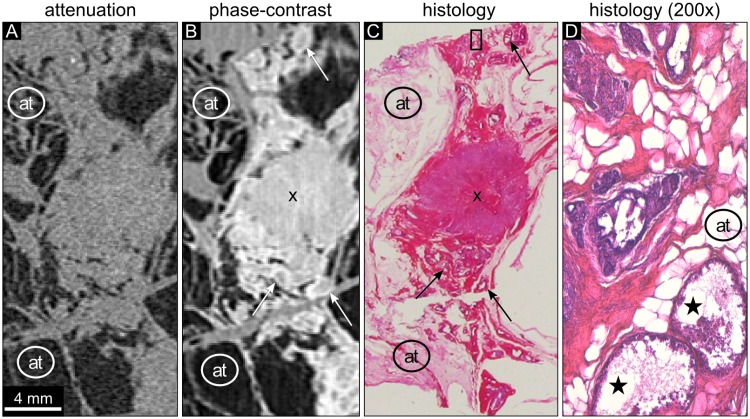
Attenuation, phase-contrast, and histology images of sample 1. (A) The absorption-based image reveals adipose tissue (at) appearing dark and areas of breast tissue. The tumor tissue cannot be further differentiated. The absorption-based image shows low contrast of all structures except for adipose tissue (at). The attenuation data is displayed in a linear range of [-60,60] HU. (B) The phase-contrast image of the same region shows a round shaped central part of the invasive tumor marked with an ‘x’ with surrounding DCIS. The bright delineation of duct walls in dilated ducts containing DCIS can be observed (arrows). (C) The histology section (HE staining) shows an invasive ductal cancer (violet, labeled with ‘x’) surrounded by DCIS and dilated mammary ducts (pink). The arrows indicate dilated ducts with intraductal carcinoma. The tumor area is embedded in an area of adipose tissue (at). (D) 200-fold magnification of the histology part indicated by the rectangle in (C) visualizes dilated ducts (violet) and an atypical epithelium that fills up completely or partially the lumen of the ducts. The DCIS areas marked by the asterisks depict central necrosis.

**Fig 2:**
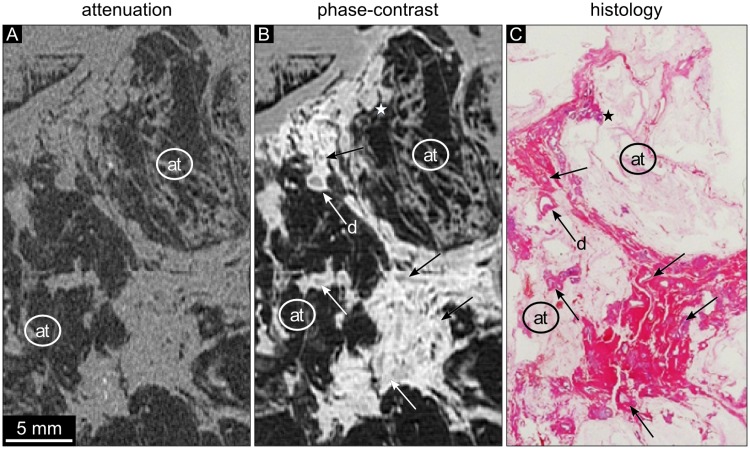
Attenuation, phase-contrast, and histology images of sample 2. (A) The absorption-based image shows low contrast of all structures except for adipose tissue (at). The attenuation data is displayed in a linear range of [-60,60] HU. (B) The phase-contrast image of the same region corresponding to (A) shows dilated ducts delineated with a bright wall. Exemplary ducts are marked by arrows. The duct labeled by the ‘d’ marks a normal ductal wall with high phase-contrast signal intensity, whereas the corresponding lumen is of lower signal intensity. In contrast, the DCIS area (asterisk and arrows) shows irregular shape of the ductal wall and lumen due to the multilayer epithelium. The phase-contrast data is displayed in a linear range of [-100,100] HUp. (C) The corresponding histological section (overview, HE stained) visualizes areas of fibrous tissue with violet ductal structures in different directions. The triangular tissue structure in the upper part of the slice represents an area of low grade DCIS (asterisk), which can also be seen in the phase-contrast image (B).

**Fig 3:**
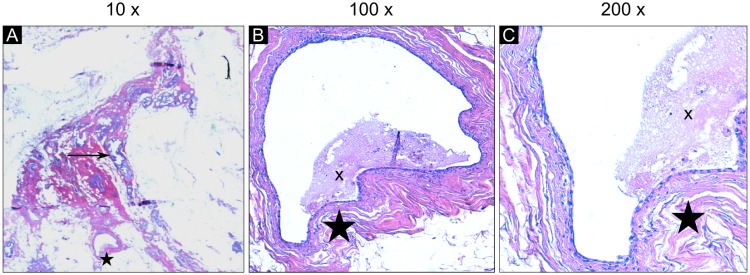
Histopathologic slices of sample 2 in different magnifications. (A) In 10-fold magnification, the details of the duct marked by the asterisk cannot be visualized. This duct is the same as depicted in the histology image in Fig 2C labeled by the ‘d’. The arrow shows an exemplary DCIS structure. (B) The 100-fold magnification visualizes the normal epithelial structure. The lumen of the ducts is partially filled by debris labeled by the ‘x’. (C) The 200-fold magnification displays the epithelial monolayer in the duct in further detail.

**Fig 4:**
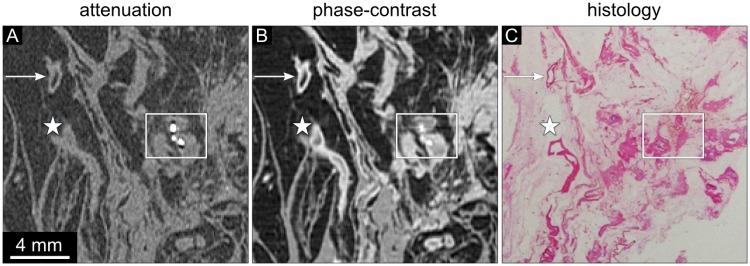
Attenuation, phase-contrast, and histology images of sample 3. (A) The absorption-based image clearly depicts microcalcifications (frame) but shows low contrast of the vessel wall (asterisk) and very little contrast of the soft tissue component of the DCIS area. The attenuation data is displayed in a linear range of [-60,60] HU. (B) The phase-contrast image visualizes a clear depiction of the vessel wall (asterisk). The calcifying DCIS region shows moderate soft tissue contrast. The phase-contrast data is displayed in a linear range of [-100,100] HUp. (C) The histological section (HE stained, overview) of sample 3 reveals a tubular structure in the left part of the section representing a vessel with a tortous segment in the lower border of the section (asterisk). Areas of calcifying DCIS can be seen in the right part of the section (frame).

Macroscopic examination of tumor 1 revealed an irregularly shaped, very stiff tumor in the central part of the mastectomy sample. Microscopy displayed a diffusely growing DCIS of maximum 6 cm diameter with a multifocal invasive carcinoma of non-specific type (NST, formerly invasive ductal carcinoma) composed by four tumor nodules of 4, 2, 0.6, and 0.2 cm of diameter, respectively. Figs 1–4 show representative slices of three tumor samples.

Sample 1 presents with an invasive carcinoma in the center and surrounding DCIS (Fig 1). The absorption-based images of sample 1 display no or very limited internal contrast differences with similar densities of ductal walls, intraductal carcinoma, and invasive carcinoma, as illustrated by way of example in Fig 1A. The phase-contrast images allow the identification of the compact invasive tumor within the surrounding ductal structures (Fig 1B). The ducts containing DCIS are delineated by bright duct walls (arrows). The histopathological slice in Fig 1C shows a clear and round shaped invasive ductal carcinoma with high cellularity and intraductal cellular components in immediate proximity. The DCIS areas are characteristically closely packed with polymorphic tumor cells within the lumen and hyperchromatic nuclei (Fig 1D). The diagnostic DCIS features within areas of dilated intramammary ducts remain hidden in the phase-contrast images due to the limited resolution of the technique in comparison to the magnification view of optical microscopy.

Sample 2 shows two areas of dilated ducts and fibrosis connected by a small semicircular tissue strand (Fig 2). In the absorption-based images (Fig 2A) dilated ducts and other soft tissue components reveal similar density with no internal contrast differences. The phase-contrast image allows clear correlation depicting the bright walls of the ducts in longitudinal and orthogonal direction (Fig 2B, arrows). The triangular tissue structure extending into the fatty tissue in the upper part of the slice represents an area of low grade DCIS (Fig 2A–2C, asterisk). Comparing the normal duct marked with ‘d’ with areas containing DCIS (Fig 2B), the phase-contrast signal intensity of the normal ductal wall does not differ much from the DCIS wall. However, the thickness of the normal duct wall is much smaller than the thickness of the DCIS and the lumen presents with relatively lower signal intensity. The DCIS containing areas have a larger epithelial layer thickness and irregular filling of the duct lumen, which increases the phase-contrast signal intensity of the DCIS areas. Fig 3 displays the histological slices of sample 2 in further detail. One can observe the widespread distribution of dilated ducts in various directions (Fig 3A, arrow). The higher magnification of the duct labeled with the asterisk visualizes a normal epithelial layer and lumen of the duct (Fig 3B & 3C, asterisk).

Sample 3 presents an area of DCIS containing microcalcifications in the right part of the slice (frame) and a vessel in the left part of the slice (asterisk) (Fig 4). The absorption image of sample 3 displays only low contrast of the vessel wall (Fig 4A). In contrast, the delineation of the vessel is well depicted in the phase-contrast images (Fig 4B). The histological section shows a vessel in the left part of the slice (asterisk) and areas of calcifying DCIS in the right part of the slice (frame) (Fig 4C). The area of DCIS is marked by a cluster of microcalcifications in both attenuation and phase-contrast images. However, the soft tissue component of the DCIS area could not be identified in the absorption-based images.

### Tumor 2

**Fig 5:**
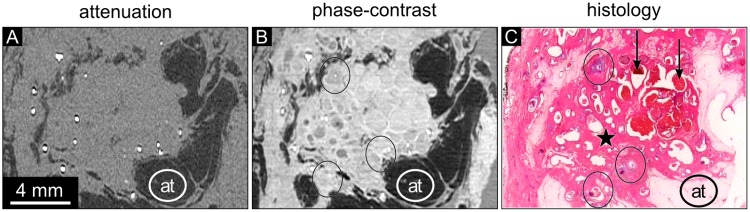
Attenuation, phase-contrast, and histology images of sample 4. (A) The absorption images reveal no differentiation of ductal structures and glandular tissue. The microcalcifications are well depicted. The attenuation data is displayed in a linear range of [-60,60] HU. (B) The phase-contrast image visualizes an overall higher signal in the haemorrhagic area but low contrast of the dilated ducts in the areas of DCIS (encircled regions). Bright delineation of duct walls is visible in both areas and the microcalcifications are clearly depicted. The phase-contrast data is displayed in a linear range of [-100,100] HUp. (C) Histological section (overview, HE stained) showing haemorrhage in an area of dilated ducts with normal monolayer epithelium (arrows) and regions of ducts with multilayer epithelium and microcalcifications representing DCIS (circles).

**Fig 6:**
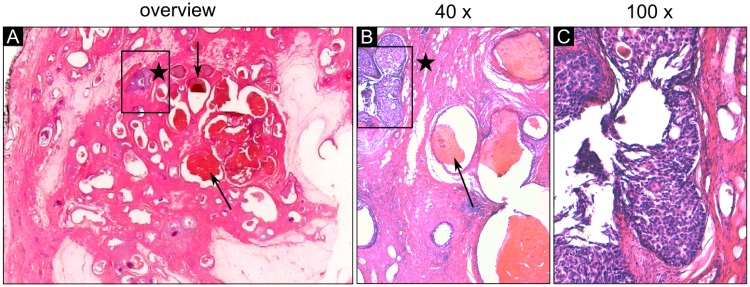
Histology slices of sample 4 in detailed magnification. (A) The histological section in overview (HE stained) indicates an area of dilated ducts with an atypical epithelium (framed violet ducts). (B) The magnification view (40 ×) clearly demonstrates dilated ducts filled up with violet stained epithelial cells (frame) and surrounding fibrous tissue (pink). These ducts filled with blood clots, which can be identified in the phase-contrast image in Fig 5B, show a normal flat monolayer epithelium. (C) The magnification view (100 ×) of the framed region reveals central necrosis within the intraductal proliferations and a high grade nuclearity of the epithelial cells showing clear evidence for the presence of DCIS.

Macroscopic examination of tumor 2 revealed a tumor nodule with an irregular shape and a whitish microcystic surface. The cysts were filled with partially yellow, partially reddish brown, crumbly material. Microscopy showed extensively fibrotic breast parenchyma containing ectatic ducts lined with a DCIS grade 3, focal intraluminal calcifications, and comedonecroses. Figs 5 and 6 show representative slices of one tumor sample.

Sample 4 consists of haemorrhage in the ducts and multiple areas containing DCIS visualized in Fig 5. The absorption image of sample 4 shows no internal soft tissue contrast within the sample section (Fig 5A). The phase-contrast image allows the depiction of the extent of the ducts in the haemorrhage area as well as in the DCIS area (Fig 5B). The calcification clusters are depicted both by phase-contrast and absorption images. The histological section of sample 4 displays an area of haemorrhage in the center of the slice (Fig 5C). Intraductal blood clots are marked by way of example by arrows. The small scattered areas of the violet stained ducts represent diffusely growing calcifying intraductal carcinoma grade 3 (circles). Magnification views of the histological section demonstrating blood filled ducts with normal monolayer epithelium and a DCIS containing area (violet) are shown in Fig 6. The flat monolayer epithelium of the normal dilated ducts filled with blood clots can be identified in Fig 6B. The intraductal proliferations filling up the duct with polymorphic tumor cells and debris indicating comedonecrosis (violet) are clearly visible using a magnification factor of 40 and 100 (Fig 6B and 6C).

## Conclusion

Complete resection in breast conserving therapy of intraductal carcinoma remains a challenge. Although specimen radiography is well established to provide accurate intraoperative margin assessment, re-excision rates are much higher in the presence of extensive intraductal carcinoma and the recurrence rate after primary surgery remains significant [6]. Phase-contrast imaging of the breast has been shown to be a promising technique for ex-vivo breast imaging outperforming the limitations of attenuation-based specimen radiography. Successful correlation with histopathology being the standard of reference in a case of intraductal carcinoma using synchrotron radiation has been described by Sztrókay et al. suggesting that the visualization of the ductal walls of dilated intramammary ducts allows the identification of areas containing DCIS [38].

In this study, we evaluated for the first time the potential of a grating-based phase-contrast computed tomography set-up using a conventional, laboratory X-ray source for ex-vivo imaging of samples containing DCIS. Our results demonstrate a successful correlation of GBPC-CT data sets with the histopathological sections of ex-vivo breast samples. Outperforming absorption-based images of the same data-set, the phase-contrast images allow the clear depiction and differentiation of both invasive carcinoma and surrounding areas of intraductal carcinoma. Even single dilated ducts, intraductal bleeding, and distinct vessels were matched correctly with the HE stained sections. In the case of invasive disease phase-contrast CT images could clearly depict areas of surrounding dilated ducts suspicious for DCIS.

However, the consensus meeting showed that identifying and classifying areas of DCIS and epithelial hyperplasia during the pathological inspection of the sections is not a sharp-edged process but a more feature collecting scan. Pathognomonic features of DCIS like atypical epithelial growth, mitoses, and nuclear size could be revealed by light microscopy with magnification factors of at least 100. These features are essential for the differentiation of dilated ducts representing either sclerosing adenosis or malignant intraductal epithelial growth.

In this regard, GBPC-CT for ex-vivo sample assessment will not be able to compete with histological work-up as long as the spatial resolution is not drastically improved, which is the major limitation of the presented method. Currently, the isotropic spatial resolution lies around 100 μm. Increasing the spatial resolution can be realized for example using X-ray detectors with smaller physical pixel sizes and a redesign of the interferometer in combination with an X-ray source with a smaller focal spot size.

A different phase-contrast approach is propagation-based phase-contrast imaging, which can also be realized with laboratory X-ray sources, as shown in a current study revealing highly detailed brain structures [43]. GBPC-CT does currently not reach this resolution in a laboratory environment, but is in general a more sensitive method and enables quantitative imaging. At synchrotron facilities, high spatial resolution can be reached with GBPC-CT, as shown in Zanette et al. [44].

In a recent breast cancer phase-contrast tomography study, which was performed with propagation-based imaging at a synchrotron facility, a high resolution match between breast tissue and histology was achieved [30]. Although the effective pixel size of our experiments performed with the laboratory GBPC-CT setup is much larger, visual comparison allows almost similar contrast.

Further, the measurement duration is still way too long for time-critical application. The sensor of the detector used here limits the efficiency, as well as the gratings on silicon substrate cause substantial unwanted beam absorption. In order to increase the time performance, more efficient detector systems, improved grating properties, and X-ray sources with higher flux would allow to reduce the duration of the GBPC-CT scan enormously.

In contrast to histopathological sections depending on distinct slices and compared to two-dimensional approaches, phase-contrast CT provides full three-dimensional capability allowing a more precise margin assessment over the whole sample especially for non-calcifying DCIS extending the invasive tumor. Thus, GBPC-CT sample imaging is able to provide essential histological landmarks and to identify suspicious areas within a sample to navigate the placement of histological sections.

In conclusion, we show that improved ductal wall assessment is feasible with grating-based phase-contrast computed tomography in a laboratory environment providing additional diagnostic benefits. Further optimization of the GBPC-CT setup will increase the potential application for specimen tomography at higher spatial resolution and in shorter measurement duration.

## Abbreviations

CT: computed tomography
DCIS: intraductal carcinoma in situ
PC-CT: phase-contrast computed tomography
GBPC-CT: grating-based phase-contrast computed tomography
NST: invasive carcinoma of non-specific type
HE staining: haematoxylin-eosin staining
